# Newly identified form of phenotypic plasticity of cancer: immunogenic mimicry

**DOI:** 10.1007/s10555-023-10087-1

**Published:** 2023-02-08

**Authors:** József Tímár, Kenneth V. Honn, Mary J. C. Hendrix, György Marko-Varga, Sirpa Jalkanen

**Affiliations:** 1 Department of Pathology, Forensic and Insurance Medicine, Semmelweis University, Budapest, Hungary; 2 Departments of Pathology, Oncology and Chemistry, Wayne State University, Detroit, MI USA; 3 Barbara Ann Karmanos Cancer Institute, Detroit, MI USA; 4 Department of Biology, Shepherd University, Shepherdstown, WV USA; 5 Clinical Protein Science and Imaging, Biomedical Centre, Department of Biomedical Engineering, Lund University, Lund, Sweden; 6 Medicity Research Laboratories, Turku, Finland; 7 InFLAMES Flagship, University of Turku, Turku, Finland

**Keywords:** Cancer plasticity, Epithelial mesenchymal transition, Vasculogenic mimicry, Megakaryocytic mimicry, Immunogenic mimicry

## Abstract

Cancer plasticity is now a recognized new hallmark of cancer which is due to disturbances of cell differentiation programs. It is manifested not only in various forms like the best-known epithelial-mesenchymal transition (EMT) but also in vasculogenic and megakaryocytic mimicries regulated by EMT-specific or less-specific transcription factors such as HIF1a or STAT1/2. Studies in the past decades provided ample data that cancer plasticity can be manifested also in the expression of a vast array of immune cell genes; best-known examples are PDL1/CD274, CD47, or IDO, and we termed it immunogenic mimicry (IGM). However, unlike other types of plasticities which are epigenetically regulated, expression of IGM genes are frequently due to gene amplifications. It is important that the majority of the IGM genes are regulated by interferons (IFNs) suggesting that their protein expressions are regulated by the immune microenvironment. Most of the IGM genes have been shown to be involved in immune escape of cancers broadening the repertoire of these mechanisms and offering novel targets for immunotherapeutics.

**Supplementary Information:**

The online version contains supplementary material available at 10.1007/s10555-023-10087-1.

## Introduction

### New hallmark of cancer: phenotypic plasticity

During the past few decades, Weinberg and Hanahan established the concept of hallmarks of cancer [1], which progressively advanced our knowledge of key genetic and phenotypic features of cancer [2]. In a recent updated version, Hanahan suggested several novel Hallmarks such as phenotypic plasticity, nonmutational epigenetic reprogramming, polymorphic microbiomes, and senescence [3]. Terminal differentiation in normal cells provides the activation of necessary factors to be able to fulfill homeostatic function, which is contradictory to continuing proliferation. In cancer, terminal differentiation is frequently blocked and phenotypic plasticity is reactivated in parallel with proliferative potentials [4]. Cancer stemness was already part of the Hallmark package [2] which may drive cancer plasticity programs. Unlocking cellular plasticity in cancer occurs in three forms: dedifferentiation (returning back to the progenitor state), blocked differentiation (sustaining progenitor features), or transdifferentiation when a differentiation program is switched into a new one, which is generally incompatible with the original cell type.

### Dedifferentiation

There are several examples regarding dedifferentiation. In colon carcinogenesis, the loss of developmental transcription factors SMAD4 [5] and HOXA5 [6] are responsible for the reappearance of stem and progenitor features and increased proliferative capabilities. In melanoma genesis, the loss of MITF, responsible for tissue specific gene expression of melanosomal proteins, results in the reactivation of neural crest progenitor genes [7]. Furthermore, the upregulation of ATF2 can also result in downregulation of MITF and dedifferentiation of melanocytes [8]. During carcinogenesis of pancreatic islet cell carcinomas upregulation of miRNA that is lost during terminal differentiation is the cause of dedifferentiation and malignant transformation [9].

### Blocked differentiation

Blocked differentiation has been observed in hematopoietic malignancies, melanomas, and hepatic and bile duct cancers. In the case of various leukemias (acute promyelocytic or myeloid versions), development of fusion genes PML-RARα and AML1-ETO, respectively [10], results in blocked terminal differentiation of myeloid precursors. These genetic traits can be exploited therapeutically either by RA or chromatin remodeling using HDAC, resulting in stimulated differentiation of myeloid progenitors [11]. In melanoma, the maintained expression of SOX10 transcription factor is responsible for blocking terminal differentiation of melanocytes [12]. In the case of intrahepatic cholangiocarcinomas, mutation of the IDH1/2 results in the production of oncometabolite D2HG, which inhibits terminal differentiation of liver progenitor cells by suppressing the expression of the HNF4a transcription factor [13].

### Transdifferentiation

Metaplasia, switching from one differentiated tissue to another one, is a well-known cancer progenitor state. Squamous metaplasia of the respiratory epithelium, adenomatoid metaplasia in the esophagus (Barret change), and intestinal metaplasia in the stomach epithelium are well-known tumor precursor lesions. However, transdifferentiation is a much more general phenomenon in various cancer types. During carcinogenesis of ductal pancreatic adenocarcinoma (PDAC), acinar cells transdifferentiate into ductal cells due to downregulation of either PTF1a [14] or MIST1 [15] transcription factors. Another regulator of ductal differentiation is SOX9 TF, the overexpression of which in acinar cells can also result in downregulation of PTF1a and MIST1 and transdifferentiation into ductal cells [16]. In prostate adenocarcinomas, expression of AR is a hallmark and serves as an efficient therapeutic target. However, upon development of androgen resistance, upregulation of SOX2 transcription factor occurs inducing neuroendocrine differentiation of prostate adenocarcinoma [17]. In lung adenocarcinoma, one of the most frequently mutated oncogenes is EGFR, and the mutated form offers a feasible therapeutic target. However, resistance to EGFR inhibitors tends to occur sooner or later during the course of the disease due to secondary resistance EGFR mutations or activation of other oncogenic signaling pathways. Interestingly, one of the resistance mechanisms in EGFR inhibitor treatments is the transdifferentiation of lung adenocarcinoma to small cell neuroendocrine cancer [18].

## Recognized forms of phenotypic plasticity

### Epithelial-mesenchymal transition (EMT)

EMT was described in embryogenesis, later in wound healing, and now a major hallmark in cancer progression. EMT defines a specific form of transdifferentiation of an epithelial cancer cell from the epithelial to mesenchymal phenotype, which is not caused by genetic alterations but regulated epigenetically—and fully reversible [19]. When observed in human cancers, epithelial (E-cadherin and cytokeratins) and mesenchymal (N-cadherin and vimentin) markers were used to define this plasticity with the help of antibodies specific for cell-adhesion molecules and intermediate filaments [20, 21]. However, in human cancer, EMT is not complete in the tumors, but cancer cells are entrapped in an intermediate state when epithelial and mesenchymal markers co-exist in the cancer cells. This is called partial EMT (p-EMT) [22]. EMT is regulated by the wild-type forms of tissue specific EMT-transcription factors (TF). The functional role of EMT in cancer is the close association with motility, invasiveness, and metastatic potential, in addition to chemoresistance [19]. EMT-TFs are SNAIL, SLUG, TWIST1/2, and ZEB1/2 [19, 22]. However, besides these core EMT-TFs, there are many other TFs which can promote EMT, like TBXT, E47, KLF4, PPRX1, GSC, RUNX1, TCF4, SIX1, FOXC2, or SOX4, and can be expressed in a tissue or cancer type specific manner [22]. There is an additional level of EMT regulation by miRs. MiR-200 is a direct repressor of ZEB-TFs; miR-34 and miR-200 are SNAI2/SLUG repressors, while miR-203 is a SNAI1 repressor [22].

These TFs are capable of repressing the expression of epithelial genes, including CDH1 and CRB3. On the other hand, TFs activate expression of mesenchymal genes such as VIM, FN1, and CDH2, but they also activate expression of proteolytic enzymes (metalloproteinases) and several cytoskeletal proteins and downregulate components of epithelial junctions [22]. EMT programs are induced by either autocrine or paracrine factors of the cancer tissue. The best-known example is TGFβ, but various growth factors, including EGF, HGF, FGF, VEGF, and IGF [23], and cytokines such as IL-8, hypoxia, mechanical ECM stress, or specific oncometabolites [24] are all powerful EMT inducers. The tumor immune microenvironment can also affect EMT programs, since FN1 and CRB3 marker genes are IFN-regulated ones (IRG = IFN-regulated gene); furthermore, some of the TFs of EMT are all regulated by IFN: SNAI1, TWIST1/2, ZEB1/2, SIX1, SOX4, and TCF4, according to the Interferome database [25] (Supplementary Table 1). Furthermore, several TFs involved in dedifferentiation, blocked differentiation, or transdifferentiation are also IRGs: HOXA5, SMAD4, MITF, ATF2, RUNX1, SOX2, and SOX9 [25] (Supplementary Table 1). The tissue-specific roles for EMT-TFs are demonstrated in several cancer types: SNAI1 was shown to be involved in breast cancer-EMT programs [26] but not in pancreatic ones, where ZEB1 has a more significant role [27]. Also noteworthy, EMT-TFs may have opposing roles in a particular tumor type, such as melanoma, where ZEB1 promotes EMT programs with the help of TWIST1, while ZEB2 in cooperation with SNAI2/SLUG are inhibitors [28].

EMT induced by EMT-TFs not only activates mesenchymal programs but also activates stem cell specific programs in cancer [19]. Furthermore, it is now evident that EMT is associated with expression of various pro-inflammatory cytokines modulating the composition of the tumor microenvironment [29, 30].

While, by definition, EMT can occur in epithelial cancers, there is now evidence that activation of EMT-TFs and their programs is not specific, because similar programs can be observed in glial tumors, neurogenic tumors, sarcomas, or even in leukemias [31].

In cancer, one can ask if there are other types of transdifferentiation in addition to EMT? Pathologists are familiar with the appearance of neurogenic markers in epithelial tumors, for example, in lung adenocarcinomas, but a similar example can be seen in breast and urothelial cancers as well. Furthermore, various cancer treatments can induce resistance in parallel with the conversion of the epithelial tumor to a neuroendocrine variant: anti-androgens induce such an alteration in prostate cancer [17], while EGFR inhibitors induce similar conversion in lung adenocarcinomas [18]. Unfortunately, the regulatory aspects are largely unknown underlying the epithelial-neurogenic transitions (ENT).

### Vasculogenic mimicry

Vasculogenic mimicry (VM) of melanoma was described in 1999 [32], now recognized as a non-angiogenic alternative for tumor tissue vascularization [33]. VM was subsequently observed in various cancer types (carcinomas of the breast, ovary, lung, prostate, and bladder), in sarcomas and CNS tumors [34], and its presence was shown to be linked to a more aggressive and metastatic phenotype. Tumor cells with VM capabilities overexpress a vast array of endothelial-associated genes and downregulate the linage-specific ones, suggestive of transendothelial differentiation [35]. Those endothelial genes are VE-cadherin (CDH5), ESM1, S1PR1, PDPN, TIE1, and EphA2. In parallel with the appearance of VM properties in melanoma, expression of pluripotent stem cell genes are also upregulated [36]. It is noteworthy that VM provides a functional perfusion pathway composed of cancer cells [37]. There are several mechanisms that can induce this transendothelial differentiation of cancer cells. ECM proteins produced by melanoma cells can induce VM: tumor cells can express and produce LN5γ2, which in return activates MMPs -1, -2, -9, 14 as well as VM [38]. Activation of vascular signaling pathways can also be involved in the induction of VM. Activation of VE-cadherin and EphA2 and their downstream signaling pathways are associated with VM involving PI3K, FAK, and ERK1/2, leading to upregulation of MMP-2 and -14, resulting in LN5γ2 cleavage [39, 40]. Although the transcription factors involved in these processes are not well known, in hepatocellular carcinoma, the VM capability involves the EMT regulator TWIST1 [41]. VEGF-A through activation of VEGFR1 was shown to activate VM programs in melanoma and ovarian carcinoma. In this case, the signal transduction pathway involved SRC together with PI3K/AKT and ERK1/2 [42]. VEGF-A in ovarian carcinoma can induce upregulation of VM genes, VE-cadherin, EphA2, matrix metalloproteinases, and MMP-2 and -9 [42]. It is also important that two inhibitors of VM have also been identified, SERPINF1 and PEDF [38]. Reactivation of stem cell signaling pathways was also observed in cancer cells expressing the VM phenotype. Expression of the embryonic morphogen Nodal in melanoma activated its receptors ALK4, -5,-7 and ACTR-IIB [43]. Moreover, it activated the downstream signaling pathway of SMAD2/3 and induced stem cell characteristics, invasion, and VM in melanoma and breast carcinoma cells [44]. It was also shown that in aggressive melanoma, Notch4 is overexpressed which is a direct inducer of Nodal expression. Last, but not least, in various cancer types (melanoma, hepatocellular carcinoma, and sarcoma), it was demonstrated that hypoxia can induce transendothelial differentiation and VM [45]. VM-associated genes VEGF-A, VEGFR1, EPHA2, TWIST, and Nodal all have HRE promoters and are responsive to HIF activation [46], while VE-cadherin and PEDF are regulated through an indirect way. Hypoxia through HIF1α can stabilize NOTCH and activate its signaling, leading to Nodal expression [47]. It is also interesting that another regulatory pathway emerged in VM, the IFN signaling, since major components of the VM phenotype (CDH5, EphA2, PDPN, S1PR1, and TIE1) are IFN regulated genes [25] (Supplementary Table 2), raising the possibility that the immune microenvironment also can induce this phenotype by the help of the STAT1/2 transcription factors.

### Megakaryocytic/platelet mimicry

Although it is much less appreciated and not well defined, the transdifferentiation of cancer cells to a megakaryocytic phenotype is also documented [48]. The platelet cell adhesion molecule, PECAM/CD31, was found to be expressed by lymphoma and leukemia cells, although this can be considered as primarily dedifferentiation. However, a systematic analysis of CD31 gene expression in various human cancer cell lines demonstrated a widespread expression at the RNA as well as protein levels [49]. *In vitro* testing also verified the functional expression of the protein by tumor cells shown to be involved in tumor cell-endothelial interactions [50, 51].

Another platelet receptor, thrombin receptor PAR1, was also found to be ectopically expressed by a variety of human cancer cell types, such as melanoma and breast and colon carcinoma [52]. The thrombin-induced signaling pathway involves PI3K, PKC, and Ca++ [53]. It is noteworthy that in melanoma, the increased expression of PAR-1 was associated with the loss of the expression of AP2 transcription factor [54].

The third membrane receptor of platelets, the αIIbβ3 integrin (CD41/61), was also found to be ectopically expressed by a wide variety of human cancer types: breast, colorectal-prostate, thyroid cancers αIIbβ3, and melanoma [55, 56]. *In vitro*/*in vivo* studies provided evidence for the functionality of expression to be involved in ECM and fibrinogen interactions, cell motility, and metastasis formation. Tumor cell αIIbβ3 integrin signals not only through FAK but also through PKC [57]. Furthermore, overexpression of αIIbβ3 in human melanoma cells resulted in increased VEGF-A and bFGF production, increased tumor-induced neoangiogenesis, and expression of some VM marker genes [58]. In bone marrow stem cells, megakaryocytic differentiation is driven by the transcription factor WT1, and αIIbβ3-positive human melanoma cells express significant levels of WT1 [48].

Activation of platelets involves the production of various arachidonic acid derivatives including prostaglandins, prostacyclins, and 12-HETE, all involved in the aggregation process. 12-HETE is produced by 12/15-LOX enzymes, which have four isoforms, ALOX12, the platelet-specific one, ALOX12B, ALOX15, and ALOX15B. Human metastatic tumor cells produce 12-HETE which is involved in tumor cell-endothelial cell interactions, cell motility, and metastasis formation [59]. Various human cancer types can ectopically express ALOX12 including breast, prostate, esophageal, gastric, and renal cancers as well as melanoma [60]. It is of note that ALOX12 was shown to be involved in the signal transduction of tumor cell αIIbβ3 integrin and the AMF receptor, upstream of PKC [57, 61]. It is also interesting that ALOX12 belongs to the IFN-regulated genes [25], suggesting a potential immune mechanism for its upregulation in cancers.

## Novel form of cancer plasticity: immunogenic mimicry

Discovery of immune checkpoints and the inhibitory activity of several members of them in lymphocytes [62, 63] initiated a plethora of studies in various cancers, which revealed that the PDL1 ligand (CD274) of the PD1 receptor is expressed by cancers and can be one of the main escape routes of immune destruction [64, 65]. PDL1 expression in cancers is not necessarily caused by genetic alterations (gene amplification) but rather is due to epigenetic upregulation [66]. Activation of the CTLA4/CD152 signaling in immune cells is another possible mechanism of immune evasion in tumors [62–66]. Recently, it was discovered that cancer cells can ectopically express CTLA4, in this case due to copy number gains (amplification) [67]. However, there may be other forms of immune escape mechanisms: a newly discovered one is the expression of the IDO1 enzyme producing kynurenin that induces T cell death [68–70]. Copy number variations (CNV) affecting CD40 and CD252 in cancers can also provide another novel excape mechanism [67, 71]. Another immune cell/macrophage gene widely expressed by human tumor cells (mostly hematopoietic ones but also by some types of solid tumors) is CD47, the activation of which triggers an anti-phagocytic mechanism induced by its ligand SIRPA/CD172a [72, 73]. Meanwhile, studies of the tumor microenvironment using markers specific to immune cells revealed that various cancers can express a series of CD markers: CD36 [74–77], CD58 [78], CD70 [79–82], CD160 [83, 84], CD276 [85], CD320 [86, 87], and CD336 [88]. Furthermore, several studies defined cancer stem cell markers [88, 89], among which immune cell markers CD90 [88–90] and CD166 [91, 92] were found to be present in various type of cancers. On the other hand, melanoma stem cells are characterized by CD20 expression [93]. Furthermore, in human melanoma tissues, the immunohistochemical expression of macrophage marker CD68 on tumor cells is a well-known phenomena which was not analyzed at genetic or protein levels [94]. Analysis of the melanoma proteome recently defined [95] indicated that the authentic CD68 protein is expressed in tumor stroma as well as in tumor tissue itself, the expression is higher in metastases as compared to primary tumors, and the tumor cell content was high in the metastases suggesting that the signal might come from tumor cells. On the other hand, proteomic analysis of human melanoma cell lines indicated that all of them expressed CD68 protein. (unpublished data)

Meanwhile, in most of these cases, CNV was not demonstrated to be responsible for such an ectopic expression; therefore, epigenetic mechanisms must be considered as a driving factor. In a recent study addressing human melanoma metastases, genome-wide CNV analysis revealed copy number gain of several CD genes including new ones: CD1a/e, CD48, CD84, CD93, CD209, CD217, and CD247, as well as known ones such as CD36, CD40, CD47, CD70, CD160, CD172, CD320, and IDO1 [96]. In all those instances, mRNA and protein expressions have been validated in an independent dataset. It is of note that the majority of these CD gene amplifications were detected in lung metastases, to a lesser extent in liver ones, and rarely observed in brain metastases, suggesting a unique clonal selection [96]. The pertinent literature on ectopic immune cell gene expressions in cancer is summarized on Table 1. It is important that only 9/26 of those genes are unique to melanoma [96]; therefore, the majority of them can be found in various common cancer types.

**Table 1:** Ectopic immune cell gene expressions in cancer cells

Mimicry gene	Normal expression	Ligand	IRG	IFN type-I	IFN type-II	CNG	Ref
CD1a	DC	Lipid Ag, TCR	+		+	+	98
CD1e	DC	Lipid Ag, TCR	+		+	+	98
CD20	B cell						95
CD36	Macrophage	TSP, collagen	+	+	+	+	74–77, 98
CD40	Macrophage, DC	CD40L	+	+	+	+	67, 98
CD47	Macrophage, T cell	CD172 TSP	+	+	+	+	72, 73, 98
CD48	B cell	CD244	+		+	+	98
CD58	Macrophage, DC	CD2/ICAM	+	+	+		78
CD68	Macrophage		+	+	+		96
CD70	T cell		+		+	+	79–82, 98
CD84	T cell	SH2D1A	+	+	+	+	98
CD90	T cell						88, 91, 92
CD93	B cell, macrophage, neutrophil	C1q	+	+	+	+	98
CD152 CTLA4	T cell, DC, macrophage	CD80, CD86	+		+	+	67
CD160	NK cell, T cell	HVEM					83, 84
CD166	T cells, monocytes	CD6					93, 94
CD172 SIRPA	Macrophage	CD47				+	72, 73, 98
CD209	DC	PAMP	+	+	+	+	98
CD217	T cell	IL-17				+	98
CD247	T cell		+		+	+	98
CD252	T cell, B cell, macrophage, DC	CD134				+	67
CD274 PDL1	Inflammatory cells	PD1	+	+	+	+	64–66
CD276 B3-H2	Inflammatory cells	TLT-2	+		+		85
CD320	DC	Cobalamin				+	86, 87, 98
CD336	NK cell	HSPG, PDGF					88
IDO1	Inflammatory cells		+	+	+	+	68–70, 98

*CNG*, copy number gain; *DC*, dendritic cell; *IRG*, IFN-regulated gene; *TSP*, thrombospondin

Particularly interesting is that out of these reported 26 immune genes, 10/26 are T cell genes, 9/26 are macrophage genes, and 8/26 are dendritic cell genes, whereas B cell or NK cell genes are much rare. It is also important that the majority of these ectopic immune cell genes are interferon-regulated ones (IRG), according to the Interferome database (17/26, 65.4%) [25]. Furthermore, all of these IRGs are regulated by type-II IFN (IFNγ) (Supplementary Table 3), and only 10/26 (38%) are regulated by type-I IFN (Supplementary Table 4), and none of our IRGs are regulated by type-III IFN. It worth mentioning that 7/26 (26.9%) of IRGs are regulated by IFNγ exclusively, including PDL1/CD274 and CTLA4/CD152 (Table 1). The IFN regulatory nature of the majority of IGM genes is suggesting that ectopic expression of those CD genes in cancer cells can be due to massive microenvironmental IFN exposures. This type of cancer cell plasticity seems to be different from the previously demonstrated forms (EMT, VM, or megakaryocytic mimicry), since in most of the cases (17/26, 65%), these are due to gene amplifications, thus being irreversible.

**Fig. 1:**
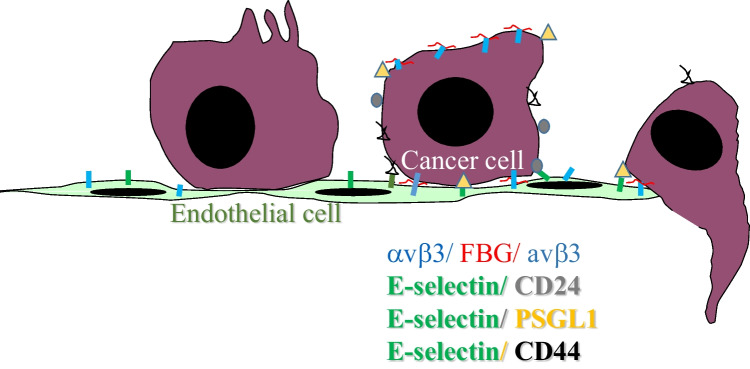
Schematic presentation of tumor cell-endothelial cell interaction during extravasation. Cancer cells express on their plasma membrane hypersialylated receptors, CD24, CD44, and P-selectin ligand-1 (PSGL-1) to dock on endothelial cell’s E-selectin. In a second step fibrinogen (FBG), receptor integrin avβ3 on both cell types stabilizes the interaction and promotes transendothelial migration

One fundamental aspect for metastasizing cancer cells is that they have acquired the ability to travel within blood and lymphatic vasculature and extravasate to various organs using a comparable multistep adhesion cascade as leukocytes do. For example, cancer cells are known to be capable of hypersialylation allowing them to create ligands for binding to E-selectin on vasculature. Such sialylated glycoprotein ligands for E-selectin expressed on tumor cells include CD44, P-selectin glycoprotein ligand (PSGL-1), and CD24 as cancer stem cell markers [88, 97, 98]. These types of aberrant interactions for non-hematopoietic cells have also clinical consequences: cancer cell interaction with E-selectin results in poor prognosis [99, 100] (Fig. 1).

Besides acquiring the trafficking capability allowing metastatic spread, the biological significance of the immunogenic mimicry could be associated with immune escape as well. Similar to the well-documented expression of PDL1 [64, 65], as well as CD47/CD172 [72, 73], CTLA4/CD152 [62, 63, 67], or IDO1 [68–70], the expression of most of these immune cell genes CD36 [74, 77], CD40 [67], CD70 [79–82], CD160 [83, 84], CD166 [91, 92, 94], CD217 [101], CD252 [67], and CD276 [85] have been documented to be involved in immune check point regulations or immune evasion. There are several known and common forms of immune evasion in cancer. Low mutation rate and consequently low amount of tumor cell neoantigens are a common theme in various cancer types [102]. Meanwhile, even with high neoantigen burden, loss of expression of HLA alleles either due to LOH (genetic factors) or epigenetic downregulation result in immune escape [103]. Furthermore, mutation and/or LOH of the beta-2 microglobulin (component of HLA class I complex) can also compromise tumor antigen presentation in cancers [104]. Another mode of immune evasion develops upon hypoxia, since VEGF is a powerful immune response inhibitor [105]. Furthermore, the immunogenic mimicry genes PDL1/CD274 and CD47 both contain HRE in their promoter region therefore can be regulated directly by HIF1A [106]. Immunotherapy is a new powerful modality of cancer management, but it is effective only in a minority of cancer patients. Selection of optimal patients for the treatments is challenging, because predictive markers are mostly lacking (see the drug indications and PDL1 accompanying diagnostics). This is due to the fact that immune checkpoint therapies are not applied in a precision manner unlike molecular target therapies. We suggest that immunogenic mimicry of cancers is a newly recognized cancer plasticity, which could have a significant role in cancer progression and immunotherapy efficacy. Therefore, it deserves significant attention. Furthermore, precise characterization of the tumor microenvironment is a special focus of molecular biologists and pathologists, which is frequently based on RNA-seq data, where the exact identification of the cell population is rarely controlled by alternative techniques [107]. Such a mimicry of cancer cells, i.e., expressing immune genes, was recently identified by analyzing single cell transcriptomes [108]. Such a widespread expression of immune cell genes in cancer cells can easily provide false interpretation of the composition of the tumor microenvironment, which can only be corrected by the immunohistochemical analysis of the tumor samples.

## Summary

**Fig. 2:**
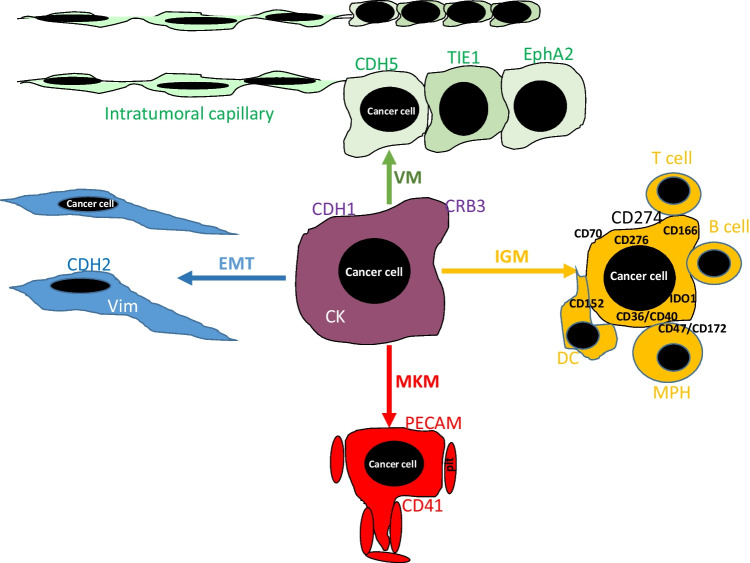
Schematic presentation of the four major forms of tumor cell mimicries: EMT (epithelial-mesenchymal transition), VM (vasculogenic mimicry), MKM (megakaryocytic mimicry), and IGM (immunogenic mimicry). Basic alterations of EMT are cadherin (CDH) switch from 1-(E) to 2-(N) and cytokeratin (CK) switch to vimentin (vim) in cancer cells. Vasculogenic mimicry (VM) involves also cadherin switch from 1-(E) to 5-(VEcadherin) as well as expression of TIE1 and EphA2 receptors on cancer cells. Megakaryocytic mimicry (MKM) involves ectopic expression of PECAM and CD41/αIIb on cancer cells promoting interactions with platelets (ptl). Immunogenic mimicry (IGM) involves ectopic expression of various immune cell genes in cancer cells, the majority of which generate inhibitory signals resulting in immune escape: CD274/PDL1, CD152/CTLA4, CD47/CD152, CD36, CD40, CD166 (ALCAM), CD276, and IDO1. DC, dendritic cell; MPH, macrophage

Cancer plasticity is a newly recognized hallmark of cancer [3] and induced or regulated not only by a vaste array of transcription factors, by the EMT-specific ones but also by less specific ones such as HIF1A or STAT1/2 (Table 2). Accordingly, cancer plasticity is involved in tumor progression and metastasis and has several forms which are involved in various steps of the metastatic cascade (Table 3 and Fig. 2). EMT is involved in several steps of the metastatic cascade from cancer initiation, cancer cell survival in various different microenvironments, local invasion, and metastatic growth. Meanwhile, EMT is mostly specific for cancer types of epithelial origin. VM occurs in a broader spectrum of cancer types, involved not only in vascularization and oxygen supply of tumor tissue but also in intra- and extravasation processes. MKM is a more specific form of cancer plasticity, again less specific for tumor types but more restricted to specific steps of the cascade, especially those involving platelet and endothelial cell interactions. We propose the novel form of cancer plasticity, immunogenic mimicry (IGM), as a part of the immune escape mechanism(s), which is involved not only in primary and metastatic tumor growth and survival but also in cancer cell survival in the circulation. Unlike the other plasticities, IGM is mostly due to genetic alterations in cancer cells and therefore is not reversible. Accordingly, after acquisition of that plasticity, it is less susceptible to microenvironmental influences in the tumor. Recognition of cancer cell plasticities and understanding the regulatory processes underlying them, offers novel therapeutic approaches, which are quite different from the current targeted therapies based on specific genetic vulnerabilities of cancer cells. Learning from the trials of the immune checkpoint inhibitor therapies, it is possible to design novel approaches targeting the IGM-type of cancer plasticity: early clinical examples are CD20 [93], CD27/CD70 [109], CD166 [110], and IDO [111].

**Table 2:** Transcription factors involved in cancer plasticities

Type of mimicry	Dedifferentiation	Ref	Blocked differentiation	Ref	Transdifferentiation	Ref	EMT	Ref	VM	Ref	MKM	Ref	IGM	Ref
Main	SMAD4 HOXA MITF ATF2	5 6 7 8	SOX10 HNF4	12 13	PTF1a MIST1 SOX9 SOX2	14 15 16 17	SNAI1 SNAI2 SLUG TWIST1 TWIST2 ZEB1 ZEB2	19	HIF1a	46			STAT1/2	Table 1
Other							TBXT E47 KLF4 PPRX1 GSC RUNX1 TCF4 SIX1 FOXC2 SOX4	22	TWIST1 SMAD2/3	41 44	AP2	54	HIF1A (CD47, CD274)	106
Probable	STAT1/2	ST1	STAT1/2	ST1	STAT1/2	ST1	HIF1a STAT1/2	22 ST1	STAT1/2	ST2	WT1 (STAT1/2)	48 #		
Cancer type	Melanoma		Leukemia Melanoma ChCA		PDAC PRCA		Epithelial Glial Sarcoma Leukemia		Melanoma Epithelial Sarcoma		Melanoma Epithelial		Melanoma Epithelial	

*EMT*, epithelial-mesenchymal transition; *IGM*, immunogenic mimicry; *MKM*, megakaryocytic mimicry; *ST*, supplementary table; *VM*, vasculogenic mimicry; *#*, see explanation in the text; *ChCA*, cholangiocellular carcinoma; *PDAC*, pancreatic ductal adenocarcinoma; *PRCA*, prostate carcinoma

**Table 3:** Involvement of various forms of cancer plasticities in the metastatic cascade

Cascade steps	EMT	VM	MKM	IGM
Primary tumor growth/survival	+	+		+
Local invasion	+	+		
LVI	+	+	+	+
Circulation/CTC survival	+		+	+
Platelet aggregation			+	
Extravasation	+	+	+	
Metastatic growth/survival	+	+		+

CTC, circulating cancer cell; EMT, epithelial-mesenchymal transition; IGM, immunogenic mimicry; LVI, lympho-vascular invasion; MKM, megakaryocytic mimicry; VM, vasculogenic mimicry

## Supplementary information

ESM 1 (DOCX 12 kb)

ESM 2 (DOCX 12 kb)

ESM 3 (DOCX 12 kb)

ESM 4 (DOCX 13 kb)

## Abbreviations

ALOX: A type lipoxygenase
AMF: Autocrine motility factor
AR: Androgen receptor
CNV: Copy number variation
D2HG: D-2-hydroxyglutarate
ECM: Extracellular matrix
EMT: Epithelial-mesenchymal transition
pEMT: Partial EMT
ENT: Epithelial-neural transition
HDAC: Histone deacetylase
HETE: Hydroxy-eicosanoic acid
HRE: HIF responsive element
IFN: Interferon
IGM: Immunogenic mimicry
IRE: IFN-regulated gene
LN: Laminin
LOH: Loss of heterozygosity
MMP: Matrix metalloprotease
MKM: Megakaryocytic mimicry
PDAC: Pancreatic adenocarcinoma
RA: Retinoic acid
VM: Vasculogenic mimicry
