# Supplementary material for: Newly identified form of phenotypic plasticity of cancer: immunogenic mimicry
Source: Cancer Metastasis Rev. 2023 Feb 8;42(1):323–34. doi: 10.1007/s10555-023-10087-1 (PMC10014767; doi:10.1007/s10555-023-10087-1)
Supplement: Supplementary file 1 — (DOCX 12 kb) [file 10555_2023_10087_MOESM1_ESM.docx]

***Supplementary Table 1. Analysis of transcription factors of disturbed differentiation and EMT in the Interferome database for IFN regulation.***

Search Conditions

Interferome Type Any

Interferome SubType Any

Treatment Concentration Any

Treatment Time Any

Vivo/Vitro Any

Species Homo sapiens

System Any

Organ Any

Cell Any

Cell Line Any

Normal/Abnormal Any

Fold Change Up 2.0

Fold Change Down 2.0

Gene Symbol List HOXA5;SMAD4;MITF;ATF2;RUNX1;SOX10;HNF4;PTF1a;MIST1;SOX9;SOX2;SNAI1;SNAI2 SLUG;TWIST1;TWIST2;ZEB1;ZEB2;TBXT;E47;KLF4 PPRX1 GSC;TCF4;SIX1;FOXC2;SOX4

Found a total of 15 Gene(s)

Ensembl Id Gene Name Description Entrez Genbank UniGene

ENSG00000176824 HOXA5 AC009951.1

ENSG00000115966 ATF2 activating transcription factor 2 [Source:HGNC Symbol;Acc:784] 1386 Hs.740955

ENSG00000187098 MITF microphthalmia-associated transcription factor [Source:HGNC Symbol;Acc:7105] 4286 BAB83926 Hs.712759

ENSG00000159216 RUNX1 runt-related transcription factor 1 [Source:HGNC Symbol;Acc:10471] 861 Hs.739683

ENSG00000126778 SIX1 SIX homeobox 1 [Source:HGNC Symbol;Acc:10887] 6495 Hs.734092

ENSG00000141646 SMAD4 SMAD family member 4 [Source:HGNC Symbol;Acc:6770] 4089 BAB40977 Hs.707908

ENSG00000124216 SNAI1 snail family zinc finger 1 [Source:HGNC Symbol;Acc:11128] 6615 CAB52414 Hs.48029

ENSG00000181449 SOX2 SRY (sex determining region Y)-box 2 [Source:HGNC Symbol;Acc:11195] 6657 CAA83435 Hs.732963

ENSG00000124766 SOX4 SRY (sex determining region Y)-box 4 [Source:HGNC Symbol;Acc:11200] 6659 CAA50018 Hs.654258

ENSG00000125398 SOX9 SRY (sex determining region Y)-box 9 [Source:HGNC Symbol;Acc:11204] 6662 CAA86598 Hs.647409

ENSG00000196628 TCF4 transcription factor 4 [Source:HGNC Symbol;Acc:11634] 6925 CBY80182 Hs.742885

ENSG00000122691 TWIST1 twist family bHLH transcription factor 1 [Source:HGNC Symbol;Acc:12428] 7291 CAA67664 Hs.66744

ENSG00000233608 TWIST2 twist family bHLH transcription factor 2 [Source:HGNC Symbol;Acc:20670] 117581 AAH17907

ENSG00000148516 ZEB1 zinc finger E-box binding homeobox 1 [Source:HGNC Symbol;Acc:11642] 6935 Hs.733308

ENSG00000169554 ZEB2 zinc finger E-box binding homeobox 2 [Source:HGNC Symbol;Acc:14881] 9839 Hs.640528
