# Supplementary material for: Newly identified form of phenotypic plasticity of cancer: immunogenic mimicry
Source: Cancer Metastasis Rev. 2023 Feb 8;42(1):323–34. doi: 10.1007/s10555-023-10087-1 (PMC10014767; doi:10.1007/s10555-023-10087-1)
Supplement: Supplementary file 2 — (DOCX 12 kb) [file 10555_2023_10087_MOESM2_ESM.docx]

***Supplementary table 2. Analysis of marker genes of vascular- or megakariocytic mimicry in the Interferome database for IFN regulation.***

Search Conditions

Interferome Type Any

Interferome SubType Any

Treatment Concentration Any

Treatment Time Any

Vivo/Vitro Any

Species Homo sapiens

System Any

Organ Any

Cell Any

Cell Line Any

Normal/Abnormal Any

Fold Change Up 2.0

Fold Change Down 2.0

Gene Symbol List VE-cadherin;CDH5;SESM1;S1PR1;PDPN;TIE1;EphA2;Nodal;Notch4;ALK;ACTR-IIB;CD31;PAR1;CD41;ALOX12

Found a total of 7 Gene(s)

Ensembl Id Gene Name Description Entrez Genbank UniGene

ENSG00000108839 ALOX12 arachidonate 12-lipoxygenase [Source:HGNC Symbol;Acc:429] 239 Hs.654431

ENSG00000179776 CDH5 cadherin 5, type 2 (vascular endothelium) [Source:HGNC Symbol;Acc:1764] 1003 Hs.76206

ENSG00000142627 EPHA2 EPH receptor A2 [Source:HGNC Symbol;Acc:3386] 1969 AAL11019 Hs.171596

ENSG00000204301 NOTCH4 notch 4 [Source:HGNC Symbol;Acc:7884] 4855 BAG57484 Hs.682718

ENSG00000162493 PDPN podoplanin [Source:HGNC Symbol;Acc:29602] 10630 Hs.468675

ENSG00000170989 S1PR1 sphingosine-1-phosphate receptor 1 [Source:HGNC Symbol;Acc:3165] 1901 AAA52336 Hs.701726

ENSG00000066056 TIE1 tyrosine kinase with immunoglobulin-like and EGF-like domains 1 [Source:HGNC Symbol;Acc:11809] 7075 AAD14299 Hs.78824
