# Supplementary material for: Newly identified form of phenotypic plasticity of cancer: immunogenic mimicry
Source: Cancer Metastasis Rev. 2023 Feb 8;42(1):323–34. doi: 10.1007/s10555-023-10087-1 (PMC10014767; doi:10.1007/s10555-023-10087-1)
Supplement: Supplementary file 3 — (DOCX 12 kb) [file 10555_2023_10087_MOESM3_ESM.docx]

***Supplementary table 4. Analysis of immunogenomic mimicry genes in the Interferome database for IFN type-I regulation.***

Search Conditions

***Interferome Type I***

Interferome SubType Any

Treatment Concentration Any

Treatment Time Any

Vivo/Vitro Any

Species Homo sapiens

System Any

Organ Any

Cell Any

Cell Line Any

Normal/Abnormal Any

Fold Change Up 2.0

Fold Change Down 2.0

Gene Symbol List CD1a;CD1e;CD36;CD40;CD47;CD48;CD58;CD70;CD84;CD90;CD93;CD152;CTLA4;CD160;CD166,CD172;CD209;CD217;CD247;CD252;CD274;CD276;CD320;CD336;IDO1

Found a total of 10 Gene(s)

Ensembl Id Gene Name Description Entrez Genbank UniGene

ENSG00000158488 CD1E CD1e molecule [Source:HGNC Symbol;Acc:1638] 913

ENSG00000090659 CD209 CD209 molecule [Source:HGNC Symbol;Acc:1641] 30835 EAW68992 Hs.278694

ENSG00000120217 CD274 CD274 molecule [Source:HGNC Symbol;Acc:17635] 29126 ABB90152 Hs.712746

ENSG00000135218 CD36 CD36 molecule (thrombospondin receptor) [Source:HGNC Symbol;Acc:1663] 948 ADI80545 Hs.736825

ENSG00000101017 CD40 CD40 molecule, TNF receptor superfamily member 5 [Source:HGNC Symbol;Acc:11919] 958 ABI49511

ENSG00000196776 CD47 CD47 molecule [Source:HGNC Symbol;Acc:1682] 961 Hs.713993

ENSG00000116815 CD58 CD58 molecule [Source:HGNC Symbol;Acc:1688] 965 EAW56654 Hs.34341

ENSG00000066294 CD84 CD84 molecule [Source:HGNC Symbol;Acc:1704] 8832 CAA11264 Hs.610260

ENSG00000125810 CD93 CD93 molecule [Source:HGNC Symbol;Acc:15855] 22918 AAB53110 Hs.708559

ENSG00000131203 IDO1 indoleamine 2,3-dioxygenase 1 [Source:HGNC Symbol;Acc:6059] 3620 AAA36081 Hs.738619
