# Supplementary material for: Newly identified form of phenotypic plasticity of cancer: immunogenic mimicry
Source: Cancer Metastasis Rev. 2023 Feb 8;42(1):323–34. doi: 10.1007/s10555-023-10087-1 (PMC10014767; doi:10.1007/s10555-023-10087-1)
Supplement: Supplementary file 4 — (DOCX 13 kb) [file 10555_2023_10087_MOESM4_ESM.docx]

***Supplementary Table 3. Analysis of immunogenomic mimicry genes in the Interferome database for IFN regulation.***

Search Conditions

Interferome Type Any

Interferome SubType Any

Treatment Concentration Any

Treatment Time Any

Vivo/Vitro Any

Species Homo sapiens

System Any

Organ Any

Cell Any

Cell Line Any

Normal/Abnormal Any

Fold Change Up 2.0

Fold Change Down 2.0

Gene Symbol List CD1a;CD36;CD37;CD40;CD47;CD48;CD58;CD70;CD73;CD83;CD84;CD90;CD93;CD152;CD160; CD166, CD172;CD209;CD217;CD244;CD247;CD252;CD274;CD276;CD320;CD336;IDO1;IDO2

Found a total of 17 Gene(s)

Ensembl Id Gene Name Description Entrez Genbank UniGene

ENSG00000158477 CD1A CD1a molecule [Source:HGNC Symbol;Acc:1634] 909 AAA51931 Hs.1309

ENSG00000090659 CD209 CD209 molecule [Source:HGNC Symbol;Acc:1641] 30835 EAW68992 Hs.278694

ENSG00000122223 CD244 CD244 molecule, natural killer cell receptor 2B4 [Source:HGNC Symbol;Acc:18171] 51744 AAD32538 Hs.157872

ENSG00000198821 CD247 CD247 molecule [Source:HGNC Symbol;Acc:1677] 919 ACC61185 Hs.669326

ENSG00000120217 CD274 CD274 molecule [Source:HGNC Symbol;Acc:17635] 29126 ABB90152 Hs.712746

ENSG00000103855 CD276 CD276 molecule [Source:HGNC Symbol;Acc:19137] 80381 AAK15438 Hs.744915

ENSG00000135218 CD36 CD36 molecule (thrombospondin receptor) [Source:HGNC Symbol;Acc:1663] 948 ADI80545 Hs.736825

ENSG00000101017 CD40 CD40 molecule, TNF receptor superfamily member 5 [Source:HGNC Symbol;Acc:11919] 958 ABI49511

ENSG00000196776 CD47 CD47 molecule [Source:HGNC Symbol;Acc:1682] 961 Hs.713993

ENSG00000117091 CD48 CD48 molecule [Source:HGNC Symbol;Acc:1683] 962 AAH30224 Hs.243564

ENSG00000116815 CD58 CD58 molecule [Source:HGNC Symbol;Acc:1688] 965 EAW56654 Hs.34341

ENSG00000125726 CD70 CD70 molecule [Source:HGNC Symbol;Acc:11937] 970 Hs.715224

ENSG00000112149 CD83 CD83 molecule [Source:HGNC Symbol;Acc:1703] 9308 CAA77755 Hs.715968

ENSG00000066294 CD84 CD84 molecule [Source:HGNC Symbol;Acc:1704] 8832 CAA11264 Hs.610260

ENSG00000125810 CD93 CD93 molecule [Source:HGNC Symbol;Acc:15855] 22918 AAB53110 Hs.708559

ENSG00000131203 IDO1 indoleamine 2,3-dioxygenase 1 [Source:HGNC Symbol;Acc:6059] 3620 AAA36081 Hs.738619

ENSG00000188676 IDO2 indoleamine 2,3-dioxygenase 2 [Source:HGNC Symbol;Acc:27269] 169355 ABM69260 Hs.676257
